# Unveiling inequality: the sociological dynamics of road infrastructure development and social justice in rural Eastern Cape, South Africa

**DOI:** 10.3389/fsoc.2024.1481133

**Published:** 2025-01-17

**Authors:** Siyabulela Christopher Fobosi, Thelma Malima

**Affiliations:** UNESCO ‘Oliver Tambo’ Chair of Human Rights, Faculty of Law, University of Fort Hare, Alice, South Africa

**Keywords:** eastern cape, rural communities, road infrastructure, social inequality, sociological implications

## Abstract

This paper examines the systemic and multidimensional impacts of inadequate road infrastructure on social inequality in the rural Eastern Cape province of South Africa, focusing on the Hlankomo community from Mdeni to Upper Tsitsana. By integrating a sociological framework, the study explores how deteriorating road conditions perpetuate structural and symbolic violence, exacerbating economic marginalization, social exclusion, and cultural disempowerment. Drawing on site observations, qualitative interviews, and photographic evidence, the research highlights how poor road infrastructure restricts access to essential services, including healthcare, education, and economic opportunities, deepening historical and spatial inequalities. The findings underscore the urgent need for transformative, equity-driven infrastructure policies that address governance inefficiencies, historical neglect, and systemic marginalization. By prioritizing inclusivity, social justice, and sustainable development, this paper contributes to the broader discourse on rural development and infrastructural justice in South Africa’s marginalized landscapes.

## 1 Introduction and background

Road infrastructure plays a critical role in enhancing the livelihoods of rural and urban communities in South Africa. Recognizing this, the South African government has implemented various programs to improve access to road networks, addressing the neglect these areas suffered during apartheid (Pillay, 2023). However, significant challenges persist, particularly in the Eastern Cape province, where the condition, volume, and connectivity of road infrastructure remain critical issues. While the rural Eastern Cape suffers from absolute poverty and marginalization, its isolation is compounded by an inadequate road network that fails to connect communities effectively to resources, services, and economic opportunities. The decline of road infrastructure in this region has historical roots dating back to the apartheid era, during which rural infrastructure, particularly in former “homelands” like the Transkei (where the Joe Gqabi District is located), was systematically underfunded and neglected. The apartheid government prioritized urban development and economic centers, leaving rural areas with minimal investment in infrastructure.

Following the democratic transition in 1994, efforts to address this historical neglect were made through national and provincial road infrastructure programs. However, these initiatives were often hampered by governance challenges, including funding shortfalls, poor planning, and a lack of technical capacity within local municipalities. In the case of the Joe Gqabi District, where rural areas such as Hlankomo, from Mdeni to Upper Tsitsana, exemplify these infrastructural deficits, the responsibility for road maintenance lies with the Eastern Cape Department of Transport and local municipalities. Despite annual budget allocations, such as the R2.5 billion earmarked for the 2022/2023 financial year (South African Human Rights Commission (SAHRC), 2023), persistent issues such as corruption, mismanagement, and rising costs of implementation have further exacerbated the backlog in road maintenance and development.

Additionally, the decentralization of road management to under-resourced municipalities has led to inconsistent oversight and implementation. Community leaders and residents frequently highlight the lack of accountability among contractors tasked with maintaining rural roads, as projects are often abandoned midway or suffer from substandard work. These systemic failures, rooted in both historical neglect and modern-day governance inefficiencies, continue to exacerbate social and economic inequalities in regions like the Joe Gqabi District. Addressing these infrastructural deficits requires a concerted effort to resolve governance shortcomings, improve accountability, and allocate sufficient resources to reverse decades of neglect.

Herbinck et al. (2023) highlight a common mismatch between government policies and rural needs across Africa but argue that South Africa’s case is unique due to the severity of its infrastructure disparities. Roads are not only poorly maintained but also insufficient in number, limiting intra- and interregional connectivity. This lack of connectivity hinders economic activities, such as transporting goods to markets, accessing healthcare, and supporting learners in reaching educational institutions. Herbinck et al. (2023) advocate for revamping government policies on rural development, with a focus on restructuring and modernizing rural economies through better-integrated road networks that facilitate movement within regions and connect them to broader economic hubs.

The importance of this paper lies in its qualitative approach, which documents the specific challenges faced by rural community members due to poor road conditions. Vijayakumar et al. (2023, p. 1039) argue that improved road infrastructure ensures labor mobility, enhances access to markets, and drives regional economic growth, ultimately addressing broader social issues such as poverty, inequality, and exclusion (Reddy, 2018). Efficient road infrastructure is especially vital for rural economies reliant on agriculture and small businesses. Mofomme (2019) notes that well-maintained transport systems improve access to markets, work, and investment opportunities, yet in the Eastern Cape, this potential is severely undermined. Nyawo and Mashau (2019a,b), cited in Pillay (2023), highlight the neglect of rural roads in South Africa, describing them as predominantly poorly maintained gravel roads that disconnect communities from essential services and economic opportunities. For example, the inability of farmers in the Eastern Cape to transport fresh produce to markets due to poor connectivity has devastating economic consequences.

The core issue addressed in this paper is how inadequate road infrastructure in the rural Eastern Cape, particularly the lack of interconnected road networks, exacerbates social inequality. Beyond addressing individual road conditions, there is a need to analyze the broader network connectivity within the Eastern Cape and how it links to other regions, fostering opportunities for trade, access to essential services, and economic integration. By documenting the socio-economic impacts of these deficiencies, this paper advocates for targeted and inclusive infrastructure development policies that prioritize both road quality and network connectivity, which are essential for fostering equitable development and improving the lives of rural residents.

Moreover, the paper underscores the need for a sociologically informed approach to infrastructure planning and policymaking that prioritizes equity, inclusion, and social justice principles. By analyzing the current state of road infrastructure in these rural areas, the research seeks to highlight the urgent need for comprehensive and sustainable infrastructural improvements that address the unique needs of rural communities. This approach is crucial for fostering an inclusive and equitable framework for understanding and addressing social inequality in South Africa’s rural landscapes. In addition, the paper explores the various forms of violence perpetuated by inadequate infrastructure, including economic violence, social violence, and cultural violence. These forms of violence manifest through limited access to essential services, isolation and disempowerment of communities, and reinforcement of hierarchical power structures. In emergencies, the lack of reliable road infrastructure can impede timely medical interventions, further exacerbating health disparities and social injustices.

Ultimately, this paper contributes to the ongoing discourse on social inequality and infrastructure development by advocating for policies and practices that promote social justice and equity. By emphasizing the critical role of road infrastructure in rural development, the research seeks to inform and influence policy decisions that can lead to meaningful improvements in the lives of rural residents, thereby fostering a more just and equitable society.

### 1.1 Research questions

- How does the inadequate road infrastructure in the rural areas of Hlankomo, from Mdeni to Upper Tsitsana in the Joe Gqabi district of Eastern Cape, South Africa, contribute to social inequality?
- What sociologically informed strategies can be implemented to promote social justice and equitable development in these communities?

The historical underdevelopment of road infrastructure during apartheid, particularly in the rural Eastern Cape, laid the foundation for ongoing marginalization. Despite post-apartheid governance initiatives, systemic barriers and governance inefficiencies continue to perpetuate socio-economic disparities. While previous studies, such as those by Ngumbela et al. (2020) and Pillay (2023), emphasize governance challenges and funding inefficiencies in rural infrastructure, they fall short of unpacking the deeper sociological implications of infrastructural neglect. This paper moves beyond existing analyzes by framing inadequate road infrastructure as a form of structural and symbolic violence that perpetuates cycles of poverty, marginalization, and exclusion in the rural Eastern Cape. Unlike prior research that primarily focuses on economic consequences, this study delves into the socio-cultural dimensions of inequality, particularly the lived experiences of affected communities in Hlankomo, from Mdeni to Upper Tsitsana. This paper represents the first phase of a broader investigation into road infrastructure challenges in the Eastern Cape. While this phase focuses on the lived experiences of rural community members, subsequent outputs will incorporate perspectives from experts such as government officials, healthcare providers, and education professionals. This expanded approach will provide a more comprehensive understanding of the governance, planning, and systemic factors influencing rural infrastructure development.

## 2 Literature review

### 2.1 Insufficient transport infrastructure

The Eastern Cape is one of South Africa’s most underdeveloped provinces, with a significant portion of its population residing in rural areas. The region faces high unemployment rates, low educational attainment, and limited access to healthcare services, all of which are exacerbated by poor road infrastructure. The absence of proper infrastructure perpetuates a state of structural violence, where systemic neglect hinders the ability of rural residents to improve their quality of life. The Eastern Cape’s poor road infrastructure reflects a legacy of historical neglect under apartheid, which prioritized urban development while systematically underfunding rural infrastructure. Ngumbela et al. (2020) note that this neglect, coupled with modern-day governance inefficiencies, including funding constraints and bureaucratic challenges, exacerbates the region’s developmental backlog. This perpetuates structural violence, as access to essential services and economic opportunities remains severely limited.

The inequalities in the geographical development of road infrastructure in South Africa can be understood through the concept of urban bias, as discussed in development literature. Urban bias refers to the disproportionate allocation of resources and development efforts to urban centers at the expense of rural areas (Lipton, 1977). Under apartheid, the urban bias was particularly stark, as infrastructure development was concentrated in cities to support industrial and economic activities while systematically neglecting rural areas, particularly former homelands like the Eastern Cape. Despite democratic governance, this legacy persists, as rural areas remain underfunded and poorly serviced, perpetuating structural inequalities.

Ngumbela et al. (2020) identify underdevelopment and poor maintenance as primary problems affecting infrastructure in the Eastern Cape. While the Eastern Cape Department of Transport (ECDOT) allocated R2.5 billion for road infrastructure in 2022/2023, achieving a 99.9% expenditure rate (South African Human Rights Commission (SAHRC), 2023), the backlog remains substantial. Funding constraints, rising implementation costs, and systemic inefficiencies continue to undermine meaningful progress. These structural limitations illustrate how infrastructural neglect is not merely a failure of governance but a perpetuation of symbolic violence against marginalized communities, reinforcing their exclusion from economic and social opportunities. While existing literature identifies the economic implications of poor road infrastructure (Bery et al., 2004; Mofomme, 2019), there remains a gap in understanding how this neglect manifests as systemic violence that undermines rural communities’ access to social justice and equity. This paper addresses this gap by integrating theoretical perspectives on structural and symbolic violence to examine the deeper societal consequences of inadequate infrastructure in the Eastern Cape.

### 2.2 Economic growth

Bery et al. (2004) argue that rural infrastructure plays a pivotal role in promoting economic growth and alleviating absolute poverty. Improved infrastructure facilitates **economic mobility**, enhances productivity, and reduces inequalities by connecting rural areas to markets and services. Their study highlights the following impacts of rural infrastructure:

1. Creating better access to employment and earning opportunities;
2. Increasing production efficiency;
3. Connecting rural communities to previously inaccessible commodities and services;
4. Saving time, which can be redirected to productive activities;
5. Enhancing the health and physical conditions of rural populations.

In the Eastern Cape, the absence of reliable infrastructure hinders these outcomes. Farmers, for instance, struggle to transport produce to markets, resulting in economic losses and perpetuating poverty. These challenges reflect structural violence, where the systemic absence of infrastructure denies communities the means to access opportunities for economic growth, while simultaneously widening existing inequalities.

### 2.3 Poverty alleviation

Khumalo (2013, p. 5644), cited in Schachtebeck and Mbuya (2016), defines poverty alleviation as efforts to combat poverty through targeted policy interventions, addressing both structural and social inequalities. In rural areas, these inequalities are compounded by inadequate infrastructure, which impedes access to critical services such as healthcare. Willie and Maqbool (2023, p. 35) argue that socio-economic issues like poverty and unemployment are exacerbated by infrastructural neglect, particularly the lack of accessible road networks. They state:

“Socio-economic issues, such as poverty levels and high levels of unemployment, are among the impediments to accessing healthcare services, which results in poor health outcomes.”

These findings align with the theory of structural violence, which explains how systemic barriers, like poor road infrastructure, deny rural communities access to fundamental rights, including healthcare and education. The exclusion of rural residents from opportunities perpetuates marginalization and reinforces social inequality.

## 3 Theoretical framework

This paper adopts structural violence (Galtung, 1969) and symbolic violence (Bourdieu, 1991) as theoretical frameworks to analyze the systemic neglect of road infrastructure in the Eastern Cape. These frameworks offer a more nuanced understanding of the infrastructural challenges compared to the modernisation theory, as they address the underlying power structures and social injustices that perpetuate inequality and marginalization.

### 3.1 Structural violence

Structural violence refers to the systemic and institutionalized barriers that prevent individuals or communities from accessing basic resources and opportunities. Galtung (1969) argues that structural violence occurs when social structures are designed in ways that harm marginalized populations by denying them their fundamental rights. In the context of the Eastern Cape, poor road infrastructure represents a form of structural violence:

- It restricts access to healthcare, education, and economic opportunities, leading to adverse social outcomes.
- It reinforces spatial inequalities, where rural communities are systematically excluded from participating in broader economic and social networks.

For example, the inability of ambulances to reach communities in emergencies or farmers’ struggles to access markets due to deteriorating roads exemplifies the systemic nature of infrastructural neglect.

### 3.2 Symbolic violence

Symbolic violence, as conceptualized by Bourdieu (1991), refers to the ways in which dominant social structures and hierarchies perpetuate inequalities by normalizing exclusion and marginalization. Inadequate road infrastructure in rural areas symbolizes the neglect of these communities, reinforcing perceptions of their lesser importance compared to urban centers. Tagarirofa (2017), cited in Pillay (2023), highlights that development initiatives in Africa often focus on rural communal values but fail to address systemic power imbalances. This neglect manifests as symbolic violence in the following ways:

- Perceived invisibility: Rural communities feel forgotten, as their needs are deprioritized in national infrastructure programs.
- Cultural marginalization: Poor road networks limit participation in cultural and social activities, weakening community cohesion and identity.

The combination of structural and symbolic violence highlights the need for a restructured policy framework that prioritizes equitable infrastructure development. Transforming road infrastructure is not just about physical improvements but also about dismantling the systemic injustices that perpetuate inequality in rural areas.

### 3.3 Relevance to rural development

While modernisation theory (Rostow, 1960) emphasizes economic growth through infrastructural development, it falls short in addressing the complex social injustices embedded in infrastructural neglect. In contrast, structural and symbolic violence theories provide a holistic framework for understanding how poor road infrastructure in the Eastern Cape perpetuates systemic inequalities. These theories emphasize that infrastructure development must be seen as a mechanism for achieving social justice, addressing both the material and symbolic needs of marginalized communities. By grounding this study in structural and symbolic violence, the paper demonstrates that infrastructural neglect is not merely a developmental issue but a reflection of deeper societal inequities. Addressing these challenges requires transformative policies that prioritize the needs of rural communities, ensuring equitable access to resources and opportunities.

## 4 Methodology

**Figure 1:**
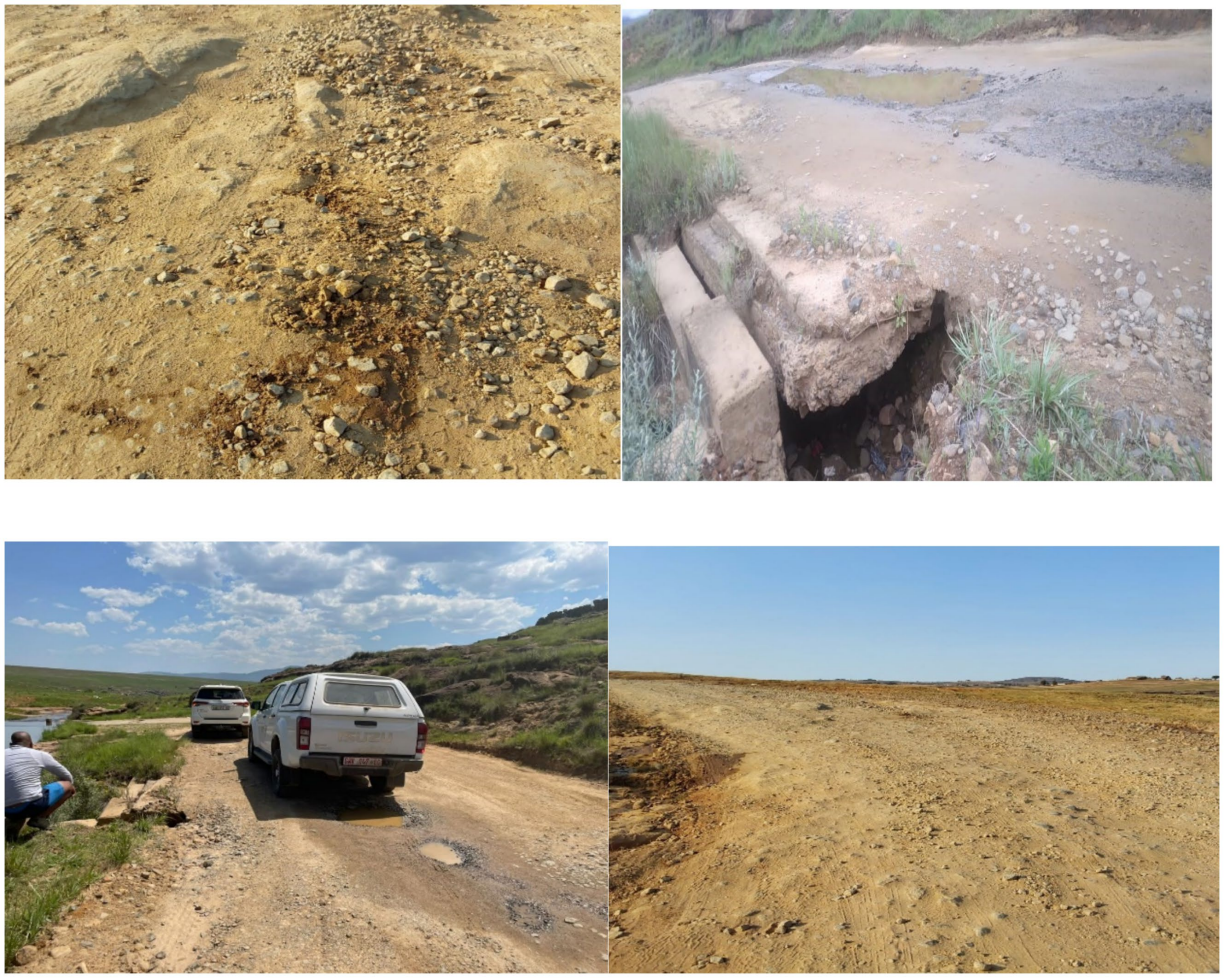
Examples of poor road conditions from Mdeni to Upper Tsitsana. Source: Author’s camera.

This paper employed qualitative research methods to investigate the impact of inadequate road infrastructure on social inequality in the rural Eastern Cape province of South Africa. The research design incorporated firsthand documentation of road conditions through site visits and the collection of photographic evidence. Site visits were conducted in the rural areas of Hlankomo, specifically from Mdeni to Upper Tsitsana in the Joe Gqabi district, to capture the diverse conditions of road infrastructure, ranging from rugged dirt paths to deteriorating asphalt. We took pictures of these conditions, as illustrated in Figure 1. This study, as the first phase of a multi-part investigation, employed a random sampling method to gather qualitative insights from residents in affected communities. While the current phase highlights the lived realities of poor road infrastructure, future outputs will include interviews with government officials, healthcare practitioners, and education professionals. These expert perspectives will offer critical insights into policy implementation, resource allocation, and the systemic challenges impacting road infrastructure development.

The qualitative methodology adopted in this study is significant because it enables a deeper exploration of the lived experiences of rural community members affected by inadequate road infrastructure. Unlike quantitative methods that may focus on statistical patterns, the qualitative approach allows for rich, narrative data that capture the nuanced realities and emotions of individuals facing infrastructural neglect. The qualitative method is particularly suitable because it prioritizes the voices of marginalized rural communities who are often excluded from formal policy discussions. Capturing their perspectives provides critical insight into how road infrastructure challenges influence access to healthcare, education, and economic opportunities, fostering a bottom-up understanding of the problem.

This study employed semi-structured interviews with residents in Hlankomo, from Mdeni to Upper Tsitsana, in the Joe Gqabi District. This method provided flexibility for participants to articulate their unique challenges while enabling the researcher to probe deeper into specific issues, such as transportation during inclement weather or its impact on emergency services. By triangulating interview data with site observations and photographic evidence, the study enhances its credibility and provides a comprehensive account of the infrastructural conditions. In addition to the empirical observations, 15 participants were randomly selected for semi-structured interviews. These interviews were conducted along a route frequently traveled by Dr. Fobosi, one of the authors, which facilitated ease of access to the participants. The semi-structured interviews provided qualitative insights into the lived experiences of rural community members, particularly how inadequate road infrastructure impacts their access to healthcare, education, and economic opportunities.

The paper also involved a comprehensive review of existing literature on infrastructure development, social inequality, and rural development in South Africa. This review encompassed academic articles, government reports, policy documents, and relevant case studies, offering insights into the historical and contemporary context of road infrastructure in rural areas. The literature review helped contextualize the empirical findings within broader theoretical frameworks and existing knowledge on the subject. The qualitative approach was chosen for its ability to provide an in-depth understanding and rich, detailed descriptions of the experiences of rural community members. This methodology facilitated the exploration of the nuanced ways in which poor road infrastructure exacerbates social inequalities.

Unlike earlier studies that highlight infrastructure challenges as isolated governance failures, this paper demonstrates that the neglect of road infrastructure reflects a broader pattern of systemic marginalization rooted in both historical inequities and contemporary policy shortcomings. By documenting the lived experiences of residents in Hlankomo, the paper highlights how inadequate roads perpetuate multiple forms of violence—economic, social, and cultural—distinguishing this analysis from previous research that primarily focuses on economic loss.

## 5 Results and discussion

This section presents the findings of the study, highlighting the socio-economic and cultural implications of inadequate road infrastructure in rural Eastern Cape communities, specifically in Hlankomo, from Mdeni to Upper Tsitsana. The results are organized into three core themes—economic impacts, educational disruptions, and healthcare challenges—demonstrating the multifaceted consequences of infrastructural neglect. These findings are grounded in interviews with community members, observations during site visits, and photographic evidence, providing a comprehensive understanding of how inadequate road networks perpetuate marginalization and inequality. The findings reveal a spectrum of road conditions that vividly illustrate the challenges faced by local communities. Through detailed site visits and photographic documentation, the paper documented the stark reality of infrastructure neglect and its profound implications for social inequality. The forms of violence examined in this context, viewed through a sociological lens, encompassed not only physical harm but also structural and symbolic violence. They include economic violence, as limited access to essential services perpetuates cycles of poverty and marginalization; social violence, as communities face isolation and disempowerment due to restricted mobility; and cultural violence, as inadequate infrastructure reinforces hierarchies of power and perpetuates inequitable social structures. This paper thus contributes to the ongoing discourse on sociologies of democracy, citizenship, and various forms of violence while advocating for a more inclusive and equitable framework for understanding and addressing social inequality in South Africa’s rural landscapes.

The socio-economic challenges faced by communities in Hlankomo must be understood through both historical and present-day lenses. The apartheid-era neglect of rural infrastructure has left a legacy of spatial inequality, which modern governance initiatives, while well-intentioned, have struggled to overcome. The 99.9% expenditure rate reported by the South African Human Rights Commission (SAHRC) (2023) highlights implementation efficiency, yet funding remains insufficient to address the massive backlog caused by decades of neglect.

### 5.1 Documentation of road conditions

The empirical investigation of road infrastructure in the rural Eastern Cape, specifically in the areas of Hlankomo from Mdeni to Upper Tsitsana, revealed a spectrum of road conditions that underscore the challenges faced by local communities. Through site visits and photographic documentation, the paper captured the stark reality of infrastructure neglect and its profound implications for social inequality.

#### 5.1.1 Types of road conditions

The roads observed ranged from poorly maintained gravel paths to severely degraded asphalt surfaces. Figure 1 depicts examples of these conditions, illustrating the extensive potholes, uneven surfaces, and lack of proper maintenance observed during the site visits from Mdeni to Upper Tsitsana. In Figure 1, the gravel road is marred by deep ruts and erosion, making travel difficult, particularly during inclement weather. These conditions not only hinder daily commutes but also restrict access to healthcare facilities and educational institutions, exacerbating social inequalities. The forms of violence are visible in the following ways:

1. **Economic violence**: Limited access to essential services perpetuates cycles of poverty and marginalization, as poor road conditions impede economic activities and opportunities. A community member shared: “I sell vegetables at the market in town, but because of the road, I sometimes cannot take my produce there. The transport costs are high, and my vegetables go bad before I can sell them. This road is killing our income.”
2. **Social violence**: Communities face isolation and disempowerment due to restricted mobility, which is evident in the difficulty of traveling on these roads, particularly in emergencies or for accessing vital services. One resident described the impact on healthcare access: “*My grandmother was very sick, and we waited hours for an ambulance that never arrived because the road was too bad. We had to carry her for kilometers to get to the main road. By then, it was too late*.”
3. **Cultural violence**: Inadequate infrastructure reinforces hierarchies of power and perpetuates inequitable social structures, as the neglect of road maintenance highlights the systemic disregard for rural communities’ needs. A local teacher explained: “*Our school is far, and many children have to walk long distances through terrible roads. This makes them miss school during the rains. It feels like we are forgotten, as if education in these areas does not matter*.”

This condition not only illustrates the physical neglect of infrastructure but also highlights the structural and symbolic violence that underpins social inequalities in these regions.

#### 5.1.2 Impacts on community life

The inadequate road infrastructure significantly affects the daily lives of community members. Interviews with local residents highlighted the challenges they face due to these conditions. Many residents expressed concerns about the safety risks posed by the deteriorating roads, particularly at night or during rainy seasons when visibility is reduced and road conditions worsen. One community member shared:

“At night, it’s dangerous to drive or walk here. The potholes are everywhere, and there are no streetlights. People are afraid of accidents or being stranded, especially during the rains.”

These safety concerns contribute to social violence as communities experience heightened vulnerability and restricted mobility. Such challenges isolate residents from economic and social opportunities, further entrenching their marginalization. The findings presented here are part of the first phase of this project, which centers on the perspectives of rural residents. Future research outputs will interrogate the perspectives of government officials and other key stakeholders to provide a holistic analysis of road infrastructure challenges. For instance, input from education professionals will further contextualize school attendance issues, while insights from government officials will illuminate the structural and policy barriers contributing to infrastructural neglect.

The findings of this study reflect the enduring impact of urban bias on road infrastructure development. While urban centers benefit from better-maintained road networks, rural communities like those in Hlankomo, from Mdeni to Upper Tsitsana, face persistent neglect. This disparity exacerbates geographical inequalities, as rural residents remain disconnected from essential services and economic opportunities.

One resident articulated this frustration: “We see new roads being built in towns while ours are left to crumble. It feels like our needs do not matter.”

This sentiment highlights how urban bias not only reinforces the physical divide between urban and rural areas but also symbolizes systemic neglect, contributing to broader patterns of marginalization.

### 5.2 Socio-economic ramifications: health and education

The socio-economic ramifications of poor road infrastructure in rural Eastern Cape are profound, particularly in terms of healthcare access and educational opportunities. Healthcare Access is significantly hindered by inadequate road infrastructure, exacerbating health outcomes for local residents. Delays in emergency medical responses due to poor road conditions pose serious risks and worsen health outcomes. Medical personnel face challenges in reaching patients promptly, which is crucial during medical emergencies. A nurse from a local clinic recounted:

“We often have to rely on community members to carry patients to the main road, sometimes on makeshift stretchers. By the time the ambulance arrives, it’s already too late in many cases.”

These barriers not only perpetuate existing health disparities but also contribute to broader social injustices within the community, as access to healthcare is a fundamental right often compromised by infrastructural neglect. Educational Opportunities are also severely impacted by inadequate road infrastructure, particularly affecting rural youth. During adverse weather conditions or due to impassable roads, students face challenges attending schools regularly. A local parent explained:

“My children have to cross flooded roads to get to school. Sometimes they do not go at all because it’s too dangerous. How can they learn properly when they miss so many days?”

This unreliable transportation infrastructure limits educational attainment and future prospects for youth in rural areas. The inability to access consistent education perpetuates cycles of disadvantage, hindering social mobility and reinforcing educational inequities among rural youth. Both healthcare access and educational opportunities are critical components of socio-economic development. A teacher from Upper Tsitsana echoed these sentiments:

“Many learners drop out because getting to school is too hard. We lose talented students every year because of these roads.”

Improving road infrastructure in the rural Eastern Cape is essential not only for enhancing mobility but also for ensuring that communities have equitable access to essential services and opportunities. By addressing these infrastructural deficiencies, policymakers can mitigate health risks, improve educational outcomes, and lay the foundation for sustainable socio-economic growth in rural communities. These efforts are crucial for fostering inclusive development and reducing disparities that undermine the well-being and prospects of residents in the Eastern Cape province of South Africa.

### 5.3 Sociological implications

The sociological implications of poor road infrastructure in rural Eastern Cape extend beyond economic consequences to encompass social exclusion and cultural impact. Economic Consequences are particularly severe, affecting agricultural activities and overall economic development. Farmers in the Eastern Cape face significant challenges in transporting their produce to commercial markets efficiently. The poor road infrastructure limits their ability to reach markets in a timely manner, thereby reducing income potential and perpetuating poverty within rural communities. This economic strain further exacerbates inequalities and hampers local economic growth.

The inadequate road infrastructure represents not only structural violence in the form of systemic barriers but also symbolic violence, as rural communities are continually marginalized. This marginalization is rooted in apartheid-era policies that deprived rural areas of critical infrastructure, a reality that present-day governance struggles to reverse. These interconnected forms of violence perpetuate socio-economic and cultural exclusion, underscoring the urgent need for targeted interventions that address both historical injustices and modern governance shortcomings.

Social Exclusion and Cultural Impact are equally pronounced in areas with inadequate road infrastructure. The isolation caused by poor roads undermines community cohesion and contributes to social exclusion. Rural residents find themselves disconnected from essential cultural and social amenities, which are often located in urban centers or accessible areas. This limitation deepens cultural inequalities, as residents in remote areas struggle to participate fully in cultural activities and events that foster community identity and cohesion.

These sociological implications underscore the multifaceted challenges faced by rural communities in the Eastern Cape due to infrastructural neglect. Beyond economic hardships, inadequate road infrastructure perpetuates social divisions and cultural marginalization. Addressing these issues requires not only infrastructure improvements but also broader societal efforts to promote inclusivity and equal access to resources and opportunities. By enhancing road networks, policymakers can facilitate greater integration of rural communities into broader economic and social networks, promoting social cohesion and cultural enrichment across the region. While earlier works (Pillay, 2023; Willie and Maqbool, 2023) focus on rural infrastructure’s economic limitations, this study expands the discourse by integrating structural and symbolic violence theories to examine the sociological dimensions of inequality. This approach reveals how infrastructural neglect systematically marginalizes rural communities, isolating them culturally and socially, in addition to exacerbating economic inequalities.

The findings of this study strongly align with the theoretical frameworks of structural violence (Galtung, 1969) and symbolic violence (Bourdieu, 1991), providing a deeper understanding of how inadequate road infrastructure perpetuates systemic inequalities in rural Eastern Cape communities.

Structural violence is evident in the systemic neglect of road infrastructure, which restricts access to essential services such as healthcare and education. The inability of ambulances to reach critically ill patients or students’ struggles to attend school due to impassable roads demonstrates how infrastructural deficits deny basic rights and perpetuate marginalization. For instance, as one resident stated:

“We have to carry patients to the main road because the ambulance cannot come here. By the time help arrives, we lose loved ones.”

This finding directly ties to structural violence by showing how institutional systems fail to address infrastructure gaps, disproportionately impacting rural communities. Symbolic violence is reflected in the perceived invisibility and exclusion of rural residents. Poor road conditions symbolize neglect, reinforcing rural communities’ marginal status compared to urban centers. As Bourdieu (1991) argued, these systemic inequalities become normalized, making rural underdevelopment seem inevitable. One teacher emphasized:

“The bad roads make us feel forgotten. It’s like education here does not matter as much as it does in towns.”

These examples show how the physical neglect of infrastructure reflects and reinforces deeper hierarchies of power, perpetuating both social and cultural marginalization.

## 6 Discussion

The discussion of this paper underscores the critical need for comprehensive improvements in road infrastructure throughout rural Eastern Cape, South Africa, to effectively address the socio-economic challenges identified. The findings reveal that neglecting infrastructure maintenance perpetuates existing social inequalities and inhibits community development. Poor road conditions not only pose safety risks but also hinder access to essential services, such as healthcare and education, which are vital for socio-economic advancement in rural areas.

Addressing Health and Safety Concerns is paramount. Effective interventions are essential to mitigate the safety risks posed by poor road conditions. Improving road surfaces, implementing erosion mitigation measures, and enhancing visibility through adequate lighting are critical steps toward ensuring the safety and well-being of rural residents. These measures not only reduce accidents and fatalities but also enhance overall community health outcomes by facilitating timely access to healthcare facilities.

Enhancing Access to Education is crucial for fostering socio-economic development. Investments in road infrastructure are essential to ensure that students can attend schools regularly, regardless of weather conditions or road quality. Reliable transportation infrastructure enables educational institutions to function effectively, supporting educational attainment and equitable access to learning opportunities for rural youth. This, in turn, contributes to breaking the cycle of poverty and empowering future generations.

Promoting Economic Development hinges on improving road conditions to stimulate growth and agricultural productivity. Enhanced infrastructure facilitates better access to markets, supports local economies, and creates opportunities for income generation among rural communities. By improving transportation networks, farmers can transport their produce more efficiently, contributing to economic stability and poverty alleviation in the region.

Fostering Social Inclusion through infrastructure improvements is crucial. By enhancing road networks, rural communities experience greater mobility and connectivity, facilitating increased interaction and equitable access to cultural and social amenities. Improved infrastructure reduces social isolation, strengthens community bonds, and fosters a sense of belonging among residents. This social cohesion is essential for promoting community resilience and collective well-being.

Policy Implications drawn from this paper emphasize the need for targeted interventions that prioritize infrastructure development aligned with community needs. Policymakers must ensure that infrastructure investments are equitable, inclusive, and supportive of long-term socio-economic development goals. By adopting policies that address infrastructure deficits and promote sustainable development, policymakers can create environments where rural communities thrive economically, socially, and culturally.

## 7 Conclusion

This paper diverges from existing literature by offering a sociological lens to examine the impacts of inadequate road infrastructure. Unlike prior studies that focus on the economic consequences of infrastructure neglect, this research highlights the intertwined manifestations of structural and symbolic violence, providing a holistic understanding of how systemic marginalization perpetuates social inequality in rural Eastern Cape communities. Therefore, this paper illuminates the profound socio-economic disparities exacerbated by inadequate road infrastructure in rural Eastern Cape, South Africa. Through a qualitative exploration of road conditions from Mdeni to Upper Tsitsana in the Joe Gqabi district, this research has documented the stark realities faced by local communities. The findings of this study underscore the long-term socio-economic impacts of apartheid-era infrastructure neglect, compounded by present-day governance challenges. The inadequate road infrastructure in the Eastern Cape perpetuates cycles of poverty, marginalization, and inequality, highlighting the need for transformative policies that address both historical and systemic inequities. Only through such an integrated approach can policymakers begin to mitigate the entrenched disparities facing rural communities. These infrastructural deficiencies not only pose safety risks and hinder emergency medical responses but also restrict educational opportunities for rural youth, reinforcing existing social inequalities. Moreover, the economic repercussions are significant, as farmers struggle to transport produce to markets efficiently, thereby limiting income potential and economic growth in the region.

From a sociological perspective, inadequate road infrastructure contributes to social exclusion and cultural marginalization, isolating communities and limiting access to cultural and social amenities. The p paper underscores the urgent need for a holistic approach to infrastructure planning and policymaking that prioritizes equity, inclusion, and social justice principles. By addressing these challenges, policymakers can foster a more just and equitable society where all residents have access to essential services, economic opportunities, and cultural enrichment. Moving forward, this research advocates for targeted policy interventions that align with community needs and promote sustainable development in rural areas. By investing in infrastructure improvements tailored to the unique challenges of rural Eastern Cape, policymakers can mitigate social inequalities, enhance community resilience, and foster long-term socio-economic prosperity.

## Data Availability

The raw data supporting the conclusions of this article will be made available by the authors, without undue reservation.
